# Prospective aquatic risk assessment for chemical mixtures in agricultural landscapes

**DOI:** 10.1002/etc.4049

**Published:** 2018-02-12

**Authors:** Christopher M. Holmes, Colin D. Brown, Mick Hamer, Russell Jones, Lorraine Maltby, Leo Posthuma, Eric Silberhorn, Jerold Scott Teeter, Michael St J Warne, Lennart Weltje

**Affiliations:** ^1^ Waterborne Environmental Leesburg Virginia USA; ^2^ Environment Department University of York Heslington York United Kingdom; ^3^ Syngenta Jealott's Hill Bracknell United Kingdom; ^4^ Bayer CropScience Research Triangle Park North Carolina USA; ^5^ Department of Animal and Plant Sciences The University of Sheffield Sheffield United Kingdom; ^6^ National Institute for Public Health and the Environment (RIVM) Centre for Sustainability Environment and Health Bilthoven The Netherlands; ^7^ Radboud University Department of Environmental Science Institute for Wetland and Water Research Faculty of Science Radboud University Nijmegen The Netherlands; ^8^ US Food and Drug Administration Center for Veterinary Medicine Rockville Maryland; ^9^ Elanco Animal Health Greenfield Indiana USA; ^10^ Centre for Agroecology Water and Resilience Coventry University Coventry West Midlands UK; ^11^ BASF SE Crop Protection–Ecotoxicology Limburgerhof Germany

**Keywords:** Risk assessment, Chemical mixture, Landscape, Agriculture, Exposure scenario

## Abstract

Environmental risk assessment of chemical mixtures is challenging because of the multitude of possible combinations that may occur. Aquatic risk from chemical mixtures in an agricultural landscape was evaluated prospectively in 2 exposure scenario case studies: at field scale for a program of 13 plant‐protection products applied annually for 20 yr and at a watershed scale for a mixed land‐use scenario over 30 yr with 12 plant‐protection products and 2 veterinary pharmaceuticals used for beef cattle. Risk quotients were calculated from regulatory exposure models with typical real‐world use patterns and regulatory acceptable concentrations for individual chemicals. The results could differentiate situations when there was concern associated with single chemicals from those when concern was associated with a mixture (based on concentration addition) with no single chemical triggering concern. Potential mixture risk was identified on 0.02 to 7.07% of the total days modeled, depending on the scenario, the taxa, and whether considering acute or chronic risk. Taxa at risk were influenced by receiving water body characteristics along with chemical use profiles and associated properties. The present study demonstrates that a scenario‐based approach can be used to determine whether mixtures of chemicals pose risks over and above any identified using existing approaches for single chemicals, how often and to what magnitude, and ultimately which mixtures (and dominant chemicals) cause greatest concern. *Environ Toxicol Chem* 2018;37:674–689. © 2017 The Authors. Environmental Toxicology and Chemistry published by Wiley Periodicals, Inc. on behalf of SETAC.

## INTRODUCTION

Many agricultural landscapes contain a mixture of crop types and/or livestock, and their management often involves the use of multiple chemicals. Many of these agrochemicals and veterinary products have the potential to move into and impact aquatic environments, resulting in potential risk from exposure to mixtures (Boxall et al. 2003; Smith et al. 2012; Schreiner et al. 2016). The detection of multiple chemicals in the environment has raised concern that current regulatory processes may be insufficient to assess the environmental risks of mixtures resulting from the use of different chemicals within agricultural landscapes (Kienzler et al. 2016).

Chemicals used in crop protection and veterinary products are highly regulated in most developed economies and undergo a standardized environmental risk assessment prior to authorization. Environmental risk assessments are always conducted on single active ingredients and may also be conducted using formulated products (e.g., European Union Regulation 1107/2009, US Federal Insecticide, Fungicide and Rodenticide Act), which can include more than one active substance as well as other chemicals such as solvents or surfactants. In addition, some countries may request the assessment of pesticide tank mixes containing more than one formulated product. Beyond these intentional mixtures, applied concurrently in time and space, there is the potential for combined exposure of aquatic environments to multiple chemicals resulting from the combination of land uses, crop types, and management practices within catchments (i.e., coincidental mixtures). A recent review of European and US regulations (Kienzler et al. 2016) concluded that intentional mixtures were well addressed through a prospective environmental risk assessment prior to approval. It also concluded that, although the potential importance of coincidental mixtures is recognized, no specific details are provided on how to assess environmental mixture effects.

Regulatory prospective environmental risk assessments calculate the risk of single compounds to aquatic organisms, generally in small edge‐of‐field water bodies with limited potential for dilution. This is a realistic worst case for single‐plant protection or veterinary medicine products but does not assess whether there is any additional risk associated with exposure to mixtures that arise from the suite of products applied to crops and/or livestock. There have been a limited number of experimental studies that have investigated the effects of a crop‐specific plant‐protection program (Van Wijngaarden et al. 2004; Arts et al. 2006). Both of these studies concluded that risk assessments based on individual compounds were sufficiently protective for these crop‐protection programs. However, environmental mixtures may also arise as a result of different chemicals applied to different targets (crops or animals) entering the water simultaneously. Other researchers have used geographic information system tools that integrate information on land use, crops, pesticide use, and other environmental data with exposure models to predict environmental exposure concentrations (Verro et al. 2002) and combined them with ecological and ecotoxicological information to assess potential risks (Sala and Vighi 2008; Solomon et al. 2013; Kapo et al. 2014). de Zwart (2005) evaluated the spatiotemporally variable net risks posed by all pesticides used in The Netherlands. Exposure was predicted using a geographic information system to identify crop types and areas, and then actual pesticide‐use data and models were used to predict drift, deposition, runoff, and drainage. The spatiotemporally variable concentrations were transformed into risk estimates using species sensitivity distributions (SSDs) and mixture toxicity modeling.

One of the key findings by de Zwart (2005) was that the ecotoxicity of environmental mixtures is generally driven by only a few compounds, a conclusion that has since been supported by empirical evidence (Belden et al. 2007; Vallotton and Price 2016). Schreiner et al. (2016) analyzed routine monitoring results for pesticides from 4532 monitoring sites across Europe and the United States. They found that mixtures were dominated by herbicides and that the most frequently detected mixtures contained 2 to 5 pesticides. These observations are highly relevant for prioritizing chemicals for management and, combined with the results of the landscape mapping and modeling studies discussed above, suggest that the assessment of environmental mixtures can be undertaken with a simplifying assumption that variations in land use can be used to estimate mixture exposure types and effects. This assumption is explored in the present study for agricultural landscapes and evaluated in more detail for multiple land uses in Posthuma et al. (2018).

In the present study we considered a mixed agricultural landscape where both plant‐protection and veterinary pharmaceutical products are used, to determine whether mixtures of chemicals pose a risk greater than that identified using existing single‐chemical or product‐based approaches. Standard agricultural scenarios, informed by case studies using real application regimes, are used to model daily exposures, which are then coupled with available effects data to assess the potential aquatic risk using a risk quotient approach for 3 taxonomic groups (i.e., fish, invertebrates, and primary producers). The magnitude and temporal pattern of potential risks were investigated and characteristics of mixtures of greatest concern identified.

Spatial scale is an important consideration in mixture risk assessment. The worst‐case assumption for judging single chemicals or products is the edge of the field because this is where exposure from spray drift, runoff, and drainage will be highest. Movement away from the edge of the field generally results in dissipation of the chemical in the water column through dilution, degradation, volatilization, and adsorption. However, when considering mixtures of chemicals, the edge of the field may not be the worst case in terms of aggregate risk; thus, a catchment‐scale (watershed) assessment should also be considered. Consideration of spatial scale should not be restricted to exposure. Protection goals may be set at the meta‐population level and thus may require a larger scale than the edge of the field, up to and including catchments, to include the range of potential nontarget species.

The present study is an output of the Society of Environmental Toxicology and Chemistry (SETAC) Pellston workshop^®^ “Simplifying Environmental Mixtures—An Aquatic Exposure‐Based Approach via Exposure Scenarios” held in March 2015, looking at 1) whether a simplified scenario‐based approach could be used to help determine whether mixtures of chemicals posed a risk greater than that identified using single chemical–based approaches, and 2) if so, what might be the magnitude and temporal aspects of the exceedances, so as 3) to determine whether the application of the approach provides insights into mixtures of greatest concern and the compounds dominating those mixtures (prioritization). The aims of the present study were to investigate these questions using standard agricultural aquatic exposure models and scenarios. Associated articles adopted the same working hypothesis to evaluate the risk of chemical mixtures from 2 other land‐use types (de Zwart et al. 2018; Diamond et al. 2018), whereas a combination of the 3 land‐use scenarios was generated to investigate these questions for catchments with different combinations of land use (Posthuma et al. 2018).

## METHODS

There are well‐established procedures for undertaking field‐scale risk assessments for plant‐protection products and, to a lesser extent, veterinary medicines. Regulatory risk assessments need to be internally consistent, so mixture‐oriented exposure estimates should be generated as much as possible using existing regulatory tools. Output from the exposure models is the daily loading of chemical to surface water summed for all relevant pathways. Agricultural chemicals are applied at discrete points in time, then dissipate in the environment, so understanding the potential for temporal co‐occurrence of contaminants in water is a central requirement for an effective mixture risk assessment.

Two exposure scenarios were developed to examine edge‐of‐field (a single‐unit scenario) and catchment‐scale (a multiunit scenario) assessments. Examples of the single‐unit scenarios are feedlots, fields, pasture, aquaculture production areas, and potentially other inputs from nonagricultural point discharges (de Zwart et al. 2018; Diamond et al. 2018), as in case study 1.

The multiunit exposure scenario is the combination of several single‐unit scenarios, including chemical and water outputs from each of the single‐unit scenarios discharging into a water body. There are 2 approaches to conducting a multiunit exposure scenario assessment. The most complex is the combination of multiple fields discharging to different locations within a catchment. This method requires hydrological characterization to appropriately model the timing of the discharges into the water body, with one or more assessment points located downstream within the catchment. A less complex method of multiunit scenario assessment assumes the simultaneous discharge of multiple field units to a water body. This latter, more conservative approach avoids the need to consider hydrology, but the estimated peaks will be higher because all discharges are to the same point in the water body and the hydrological travel time of chemicals is ignored. The present study applies this second, more conservative approach to a multiunit exposure scenario in case study 2. A more detailed discussion on field‐scale and catchment‐scale assessment and exposure scenarios is provided in the Supplemental Data.

### Case study 1: Assessment at the unit of a single field—winter wheat in the United Kingdom

#### Problem formulation

This case study addresses the following question: Is there any additional risk associated with exposure of the aquatic environment to mixtures that arise from the suite of plant‐protection products applied to a crop that would not be identified using single‐chemical assessments?

The risk for a single crop is expected to be greatest at the edge‐of‐field scale where there is limited potential for dilution and degradation within the receiving water body. A single‐field unit was modeled assuming a single crop comprising winter wheat in the United Kingdom. The case study is intended as proof of concept and not as a regulatory risk assessment, although exposure estimates are generated using an existing regulatory modeling framework for consistency with current practice. Furthermore, regulatory risk assessment at the European Union level is based on single substances, whereas at the member state level it is on a product basis. Products can contain more than one active substance, and there is often some assessment of combined risk. Although in these case studies some active substances would have been applied together as a single product, the assumption is that the assessments were done at the single‐substance level for any comparisons with the mixture.

#### Approach to exposure assessment

Pesticide risk assessments are based on either individual active substances or coformulated mixtures of active substances applied to the crop. Pesticide usage data for the United Kingdom are collected on a biannual basis (Garthwaite et al. 2013). Data for a single agricultural season (2009–2010) were obtained for a large arable farm in eastern England. There were 16 fields cultivated with winter wheat, and all fields were treated with the same suite of 13 active substances. Dates of application and actual rates were available (Supplemental Data, Table S2), so the risk assessment pertains to real conditions of use rather than the maximum label usage normally considered in prospective regulatory assessments.

The FOCUS Surface Water Scenarios (FOCUS 2001) provide a consistent framework for assessing risks to the aquatic environment from pesticides in European regulatory procedures. Ten scenarios cover the broad conditions of agriculture across Europe in terms of soils, weather, cropping, and field‐edge surface water bodies. Spray‐drift inputs to water are based on an analysis of a large database of drift experiments (Rautmann et al. 2001). The models PRZM (Suárez 2005) and MACRO (Larsbo and Jarvis 2003) simulate the fate of pesticides in soil and generate estimates of water and pesticide emissions via surface runoff and drainage, respectively. Outputs from these models and the spray‐drift calculator are inputs to TOXSWA (Beltman et al. 2006), which simulates the fate of pesticides in surface water, generating aquatic predicted environmental concentrations (PECs). While the FOCUS exposure models give PECs for water column, porewater, and sediment, we focused on water column for this case study.

One FOCUS scenario (i.e., R1 runoff) that is directly applicable to UK agricultural conditions was used to generate exposure estimates. This scenario uses a range of crop types including winter cereals and has been identified as having primary relevance to the UK agricultural situation, particularly in southeastern England (FOCUS 2001). Standard regulatory modeling procedures set out by FOCUS (2001) were followed except for 3 deviations. First, actual dates and rates of application were used as input. Second, FOCUS modeling normally relies on preassessment of pesticide application date against a 20‐yr weather data set to select a worst‐case 100‐d profile (i.e., rainfall occurring soon after application). This means that pesticides with different application dates will often be assessed with different sections of the long‐term weather data set. To overcome this, all simulations were run with the full 20‐yr series of daily weather data, and inputs to the stream were integrated using the STEPS1234 model (Klein 2007) to generate a long‐term profile of exposure concentrations. It was assumed that the same set of substances were applied in each of the 20 yr. This ensures that the assessment of exposure was conducted under a range of weather conditions and that simulations for different pesticides are consistent. Finally, only standard laboratory studies to generate environmental fate parameters for modeling were used, to ensure consistency between the different chemicals. No use was made of higher‐tier data, such as the generation of soil degradation half‐lives from field dissipation studies. Additional details are provided in the Supplemental Data.

#### Risk characterization

For each of the 13 active substances (Table 1), aquatic ecotoxicology data were taken from their respective European Union review report or the European Food Safety Authority's conclusion to calculate a regulatory acceptable concentration (RAC). The RAC is the effects assessment endpoint expressed in terms of a permissible concentration in the environment that is directly used in the risk assessment by comparing it to the appropriate field‐exposure estimate (Brock et al. 2010; European Food Safety Authority 2013a). If the RAC is not exceeded, the environmental effects of a chemical are assumed to be acceptable and low risk is concluded. We calculated RACs using the methodology of the European Food Safety Authority's (2013b) aquatic guidance. Risk to primary producers (algae and macrophytes) and acute and chronic risk to fish and aquatic invertebrates were calculated separately. If higher‐tier ecotoxicity data were available, they were also used, using the endpoints generally as presented in the respective European Union assessments and following current guidance (European Food Safety Authority 2013b). These higher‐tier data included additional species tests and aquatic micro‐/mesocosm studies for primary producers and invertebrates. The ecotoxicity data for the different taxonomic groups are presented in Table 1.

**Table 1:** Effects data and regulatory acceptable concentrations (micrograms per liter) for UK wheat case study

		Primary producers					Invertebrates										Fish										
Active ingredient	Group	Tier 1	AF	Higher tier	AF	RAC	Acute tier 1	AF	Acute higher tier	AF	Acute RAC	Chronic tier 1	AF	Chronic higher tier	AF	Chronic RAC	Acute tier 1	AF	Acute higher tier	AF	Acute RAC	Chronic tier 1	AF	Chronic higher tier	AF	Chronic RAC	Reference
Boscalid	F	1340	10			134	5330	100			53.3	1310	10			131	2700	100			27	125	10			12.5	1
Chlorothalonil	F	9.6	10	30^b^	3	10	84	100	30^b^	3	10	8.5	10	30^b^	3	10	38	100	15 (HC5)^c^	9	1.7	3	10			0.3	2
Cypermethrin	I	>100	10			10	0.3	100	0.05^b^	3	0.017	0.04	10	0.05^b^	3	0.017	2.8	100			0.028	0.03	10			0.003	3
Epoxiconazole	F	13.8^a^	10			1.38	8690	100			86.9	62.5	10			6.25	3140	100			31.4	10	10	30^f^	10	1.0	4
Flufenacet	H	2.43	10	12^b^	3	4	30 900	100			309	3260	10			326	2130	100			21.3	200	10			20	5
Fluoxastrobin	F	350	10			35	60.4	100			0.64	0.61	10			0.061	435	100			4.35	28.6	10			2.86	6
Iodosulfuron‐methyl‐sodium	H	0.83^a^	10			0.083	>10^5^	100			1000	10^4^	10			1000	>10^5^	100			1000	10^4^	10			1000	7
Mesosulfuron‐methyl	H	0.62^a^	10			0.062	>10^5^	100			1000	1800	10			180	>10^5^	100			1000	32 000	10			3200	8
Pendimethalin	H	6	10	5^b^	3	1.67	147	100			1.47	14.5	10			1.45	196	100			1.96	6.3	10	32^e^,^f^	10	0.63	9
Prochloraz	F	>32	10			3.2	770	100	1820^e^	100	18.2	22.2	10			2.22	1200	100	1340	100	13.4	24.9	10			2.49	10
Proquinazid	F	250	10			25	287	100			2.87	1.8	10			0.18	349	100			3.49	3	10			0.3	11
Prothioconazole	F	2180	10			218	1300	100			13	560	10			56	1830	100	3870	100	38.7	308	10			30.8	12
Pyraclostrobin	F	>843	10			84.3	16	100	8^b^	3	2.7	4	10	8^b^	3	2.7	6	100	4.6 (HC5)^d^	3	1.53	2	10			0.2	13

^a^ *Lemna*, others based on green algae.

^b^ Mesocosm.

^c^ Acute 96‐h median lethal concentration 5% hazard concentration from species sensitivity distribution of 11 species.

^d^ Acute 96‐h no‐observed‐effect concentration 5% hazard concentration from species sensitivity distribution of 7 species.

^e^ Geometric mean.

^f^ Higher‐tier no‐observed‐effect concentration for use against predicted‐effect concentration maximum only.

References: 1 = Boscalid SANCO/3919/2007‐rev.5 21 January 2006; 2 = chlorothalonil SANCO/4343/2000 final (revised) 28 September 2006; 3 = cypermethrin SANCO/4333/2000 final 15 February 2005; 4 = European Food Safety Authority Scientific Report (2008) 138, 1‐80; 5 = flufenacet 7469/VI/98‐Final 3 July 2003; 6 = fluoxastrobin European Food Safety Authority Scientific Report (2007) 102, 1‐84; 7 = iodosulfuron SANCO/10166/2003‐Final 3 July 2003; 8 = mesosulfuron‐methyl PPDB University of Hertfordshire; 9 = *EFSA J* 2016; 14, 4420; 10 = *EFSA J* 2011; 9:2323; 11 = *EFSA J* 2009; 7:1350; 12 = European Food Safety Authority Scientific Report (2007) 106; 13 = pyraclostrobin SANCO/1420/2001‐Final 8 September 2004, DAR 2001.

AF = assessment factor; F = fish; H = human; I = invertebrate; HC5 = 5% hazard concentration; RAC = regulatory acceptable concentration.

The RACs for primary producers and acute and chronic risks for fish and aquatic invertebrates were compared to the PECs produced by the model to give a risk quotient (RQ = PEC/RAC) for each predicted daily chemical concentration, with RQ <1 indicating acceptable risk on a per‐chemical basis. The RQ values for mixtures were calculated by summing the derived RQs of the 13 individual compounds for each day. This approach assumes concentration addition and estimates the daily total aquatic risk from all of the pesticides applied in the wheat field. Following the guidance, chronic fish and chronic invertebrate risk assessments were refined using 7‐d time‐weighted average (TWA) concentrations rather than the daily concentrations (European Food Safety Authority 2013b).

It is often observed in risk assessments of defined chemical mixtures that the risk is driven by 1, 2, or only a few chemicals (e.g., de Zwart 2005; Backhouse and Karlsson 2014). A useful method of expressing how mixture risk is characterized is the maximum cumulative ratio (MCR) approach of Price and Han (2011). The MCR is given by the sum of individual RQ values for each chemical (∑RQ) in the mixture divided by the maximum RQ within that mixture.

The MCR was calculated for each time step (i.e., daily). Following the methods of Price et al. (2012), combined exposures were grouped into categories to facilitate risk assessment and risk management. Group I contains combined exposures where one or more chemicals are of concern because they have an individual RQ >1. Group II contains combined exposures where the ∑RQ <1, and consequently these exposures are of low concern. Group III contains combined exposures where ∑RQ is > 1 only by summing the chemicals; no individual chemical has RQ >1. Group IIIA: The MCR is <2; that is, the majority of the toxicity is from one chemical. Group IIIB: The MCR is >2; that is, the toxicity is not dominated by a single chemical. Group IIIB is where the model used for mixture toxicity is most important and where further refinement based on mode of action may be important.

#### Results for case study 1

Table 2 gives the number of days when the RQ exceeded 1 for individual chemicals for primary producers and acute and chronic risk to aquatic invertebrates and fish, together with the number of days where ∑RQ across all of the chemicals exceeded 1 for each group. Table 3 translates these results into MCR categories. Table 2 also includes information on the duration of ∑RQ exceedances expressed as the number of times the ∑RQs exceeded 1 for a consecutive sequence of days (e.g., for 2, 3, 4, or 5 d consecutively), as well as the longest duration of ∑RQ exceedance.

**Table 2:** Number and percentage of total days when individual chemicals risk quotient (RQ and ∑RQ were >1 in the UK edge‐of‐field scale case study, together with the maximum RQ and consecutive days exceeding 1

	Primary producers			Invertebrate acute			Invertebrate chronic			Invertebrate chronic refined			Fish acute			Fish chronic			Fish chronic refined		
	Days RQ >1			Days RQ >1			Days RQ >1			Days RQ >1			Days RQ >1			Days RQ >1			Days RQ >1		
	No.	% Total	Max. RQ	No.	% Total	Max. RQ	No.	% Total	Max. RQ	No.	% Total	Max. RQ	No.	% Total	Max. RQ	No.	% Total	Max. RQ	No.	% Total	Max. RQ
Boscalid	0	0.00	0.01	0	0.00	0.04	0	0.00	0.01	0	0.00	0.00	0	0.00	0.07	0	0.00	0.16	0	0.00	0.03
Chlorothalonil	0	0.00	0.27	0	0.00	0.27	0	0.00	0.27	0	0.00	0.10	9	0.12	1.59	39	0.52	9.00	37	0.49	3.49
Cypermethrin	0	0.00	0.00	17	0.23	1.67	17	0.23	1.67	0	0.00	0.55	1	0.01	1.02	263	3.50	9.48	148	1.97	3.14
Epoxiconazole	0	0.00	0.76	0	0.00	0.01	0	0.00	0.17	0	0.00	0.05	0	0.00	0.03	1	0.01	1.05	0	0.00	0.29
Flufenacet	2	0.03	1.07	0	0.00	0.01	0	0.00	0.01	0	0.00	0.00	0	0.00	0.20	0	0.00	0.21	0	0.00	0.06
Fluoxastrobin	0	0.00	0.01	0	0.00	0.30	47	0.63	3.16	0	0.00	0.95	0	0.00	0.04	0	0.00	0.07	0	0.00	0.02
Iodosulfuron‐methyl	0	0.00	0.80	0	0.00	0.00	0	0.00	0.00	0	0.00	0.00	0	0.00	0.00	0	0.00	0.00	0	0.00	0.00
Mesosulfuron‐methyl	14	0.19	5.46	0	0.00	0.00	0	0.00	0.00	0	0.00	0.00	0	0.00	0.00	0	0.00	0.00	0	0.00	0.00
Pendimethalin	0	0.00	0.82	0	0.00	0.93	0	0.00	0.95	0	0.00	0.37	0	0.00	0.70	123	1.64	2.18	0	0.00	0.85
Prochloraz	0	0.00	0.15	0	0.00	0.03	0	0.00	0.22	0	0.00	0.05	0	0.00	0.04	0	0.00	0.19	0	0.00	0.05
Proquinazid	0	0.00	0.00	0	0.00	0.00	0	0.00	0.07	0	0.00	0.02	0	0.00	0.00	0	0.00	0.04	0	0.00	0.01
Prothioconazole	0	0.00	0.00	0	0.00	0.00	0	0.00	0.00	0	0.00	0.00	0	0.00	0.00	0	0.00	0.00	0	0.00	0.00
Pyraclostrobin	0	0.00	0.00	0	0.00	0.01	0	0.00	0.01	0	0.00	0.00	0	0.00	0.01	0	0.00	0.11	0	0.00	0.04
∑RQ	63	0.84	7.00	111	1.48	2.45	223	2.97	5.06	13	0.17	1.46	43	0.57	2.94	353	4.69	18.86	364	4.84	6.15
Max. duration ∑RQ > 1 (days)	3			3			3			4			3			4			14		
Days ∑RQ >1 for >1 d	13			15			29			7			8			47			300		
Days ∑RQ >1 for >2 d	2			3			3			3			2			8			240		
Days ∑RQ >1 for >3 d	0			0			0			1			0			1			187		
Days ∑RQ >1 for >4 d	0			0			0			0			0			0			140		

**Table 3:** Number and percentage of days that mixture toxicity was classed as groups based on maximum cumulative ratio categories

	Group I	Group II	Group IIIA	Group IIIB
	(single chemicals have RQ >1)	(∑RQ <1)	(∑RQ >1, no single chemical RQ >1)	
Taxonomic group			MCR <2	MCR >2
UK case study—edge‐of‐field scale wheat				
Primary producers	16 (0.21%)	7456 (99.16%)	20 (0.27%)	27 (0.36%)
Invertebrate acute	17 (0.23%)	7408 (98.52%)	76 (1.01%)	18 (0.24%)
Invertebrate chronic	64 (0.85%)	7296 (97.03%)	41 (0.55%)	118 (1.57%)
Invertebrate chronic refined	0 (0.00%)	7506 (99.83%)	8 (0.11%)	5 (0.07%)
Fish acute	10 (0.13%)	7476 (99.43%)	12 (0.16%)	21 (0.28%)
Fish chronic	282 (3.75%)	7166 (0.95%)	15 (0.20%)	56 (0.74%)
Fish chronic refined	163 (2.17%)	7155 (95.16%)	137 (1.82%)	64 (0.85%)
US case study—catchment‐scale corn and beef				
Primary producers	815 (7.44%)	9857 (89.96%)	268 (2.45%)	17 (0.16%)
Invertebrate acute	113 (1.03%)	10 844 (98.97%)	41 (0.37%)	3 (0.03%)
Invertebrate chronic	49 (0.45%)	10 133 (9.25%)	307 (2.80%)	468 (4.27%)
Fish acute	47 (0.43%)	10 908 (9.96%)	2 (0.02%)	0 (0.00%)
Fish chronic	1556 (14.2%)	8977 (81.93%)	416 (3.80%)	8 (0.07%)

MCR = maximum cumulative ratio; RQ = risk quotient.

For primary producers, only mesosulfuron‐methyl and flufenacet individually had RQs which exceeded 1, on 14 and 2 d, respectively, with maximum values of 5.46 and 1.07, respectively. The MCR group I had 16 d where an RQ of 1 was exceeded by individual chemicals, out of a total of 63 d where ∑RQ was >1. While not exceeding an RQ of 1, epoxiconazole, iodosulfuron‐methyl, and pendimethalin, in particular, contributed to occasions where ∑RQ exceeded 1 in MCR group III.

For acute risk to invertebrates, cypermethrin was the only chemical where the individual RQ exceeded 1 (maximum 1.67), which was the case for 17 d out of a total ∑RQ exceedance of 1 for 111 d. Of the 94 d in group III, indicating a mixture risk, the majority were in group IIIA, indicating the dominance of cypermethrin as the risk driver (Table 3); however, significant contributions to ∑RQ also came from pendimethalin, fluoxastrobin, and chlorothalonil.

For chronic risk to aquatic invertebrates, only fluoxastrobin and cypermethrin exceeded RQs of 1, on 47 and 17 d, respectively, and with maxima of 3.16 and 1.67, respectively. Unlike some of the other chemicals, which had refined effects assessment information, there were no higher‐tier data available for fluoxastrobin. There was a total of 159 d in group III, indicating a potential mixture risk, with the majority of those days in group IIIB. Pendimethalin and to some extent chlorothalonil, epoxiconazole, and prochloraz made significant contributions to ∑RQ. When refined using a 7‐d TWA exposure, the number of exceedances was reduced and there were no days where single‐chemical RQs exceeded 1 and only 13 d (0.17% of total days) where ∑RQ exceeded 1.

There were very few exceedances of an RQ of 1 for single chemicals for acute risk to fish. Only chlorothalonil and cypermethrin RQs exceeded 1 for 9 and 1 d, respectively, at maxima of 1.59 and 1.02, respectively. Pendimethalin made a significant contribution to ∑RQ, resulting in a total of 43 d where ∑RQ was >1, with 33 d in group III, split as 12 d in IIIA and 21 d in IIIB.

The RQs for chronic risk to fish exceeded 1 for cypermethrin, chlorothalonil, pendimethalin, and epoxiconazole on 263, 39, 123, and 1 d, respectively, with maximum values of 9.48, 9.0, 2.18, and 1.05, respectively. Group III had 71 d, with 56 in group IIIB, and with the majority of the contribution to the RQ coming from the aforementioned compounds. When refined with a 7‐d TWA, the magnitude of the RQs was significantly reduced; and for pendimethalin, all of the RQs became <1. For cypermethrin and chlorothalonil, there was some reduction in the number of days where RQs exceeded 1; but the change was not as large, which is explained by the magnitude of the RQs for those compounds. The concentration is effectively spread across a number of days when using a TWA concentration, resulting in some days exceeding an RQ of 1 using a 7‐d TWA where they previously did not when based on the modeled concentration for just that day. This is illustrated by the large increase in the number of times the ∑RQ exceeded 1 for a set of consecutive days and by the increase in longest duration of ∑RQ > 1 (Table 2). In these runoff scenarios, exposures are typically short and thus probably warrant further investigation of the potential for chronic effects on fish from short‐term exposures.

The longest duration of exceedances (∑RQ > 1) was 3 or 4 d across all taxa other than refined chronic fish, and the number of days where ∑RQ > 1 consecutively for more than 2 d ranged from 2 to 8 across taxa. For refined chronic fish (using the 7‐d TWA), the longest duration of ∑RQ > 1 was 14 d, with 240 d when ∑RQ > 1 consecutively for more than 2 d. Full results are presented in Table 2.

**Figure 1:**
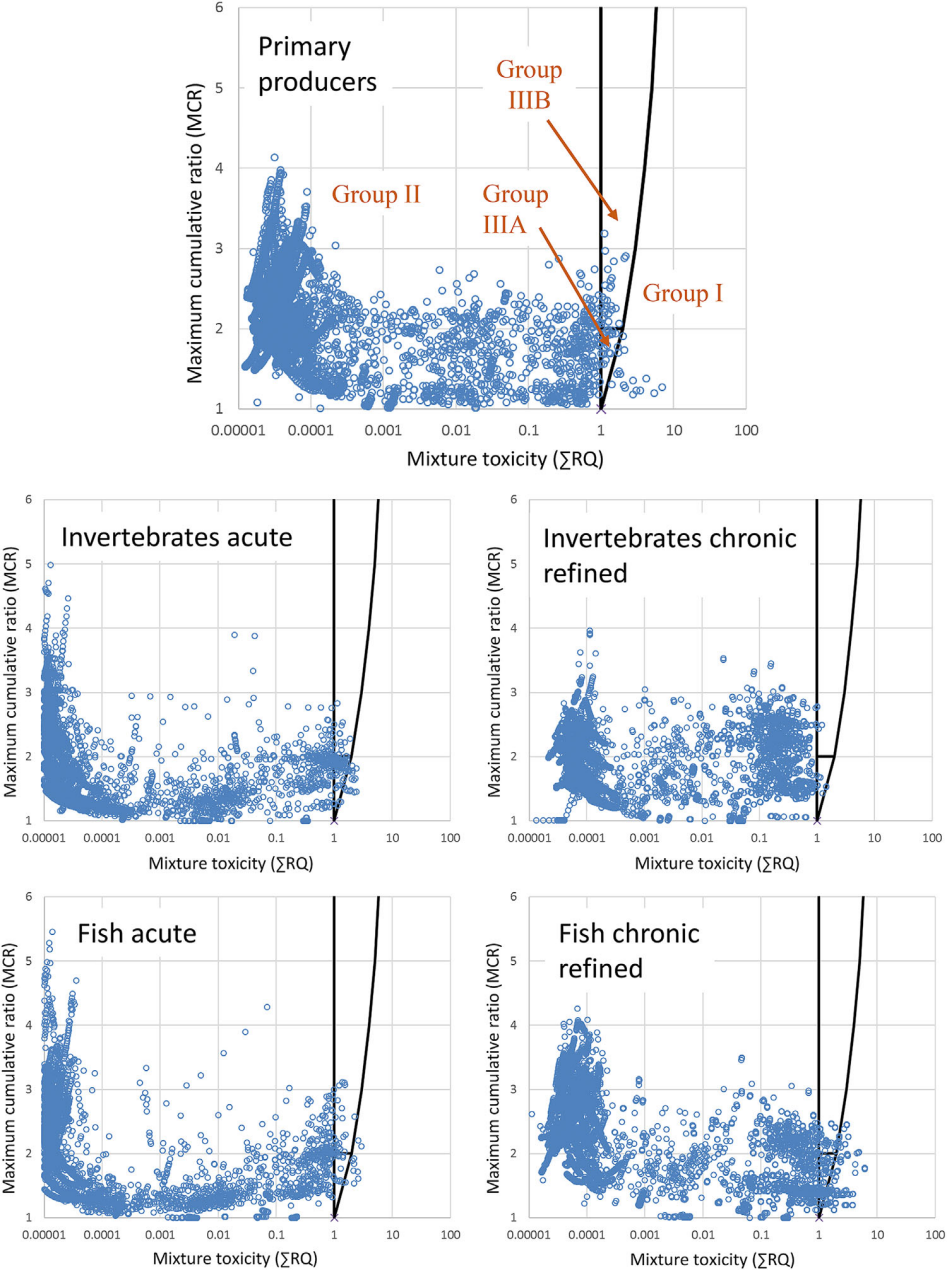
Plots of daily mixture toxicity (sum risk quotient [∑RQ], *x* axis) and maximum cumulative ratio (*y* axis) for the simulated exposure scenario of 13 plant protection products applied to a single UK wheat field over 20 yr. Group I is comprised of mixtures where individual chemicals present a risk. Group II is comprised of mixtures with no risk identified. Groups IIIA (majority of risk is driven by a single substance) and IIIB (potential risk is driven by multiple components) are comprised of mixtures where only the combined effect indicates a risk. Plots are shown for primary producers (algae and aquatic plants), aquatic invertebrates (acute and 7‐d time‐weighted average [TWA] chronic), and fish (acute and 7‐d TWA chronic).

Figure 1 graphically presents the daily predicted mixture toxicity values over 20 yr for each of the taxonomic groups assessed. The topmost chart for primary producers contains the labeled MCR groups using the categories of Price and Han (2011).

### Case study 2: Assessment at the small catchment unit scale—US corn together with cattle grazing and feedlot operations

#### Problem formulation

Agricultural fields do not exist in isolation within the agricultural landscape. The landscape consists of fields with different uses, for crops, pasture, and animal husbandry. All have potential chemical inputs into the aquatic environment. This case study addresses the following question: Is there any additional risk associated with exposure of the aquatic environment to mixtures that arise from a suite of plant‐protection products and veterinary medicines within the same catchment (watershed) that would not be identified using a single‐chemical assessment?

The risk assessment represents multiple sources of chemical inputs associated with a scenario of corn production in Iowa, USA. It considers input from crop‐protection activities together with veterinary pharmaceutical inputs from use in beef cattle from 3 runoff sources: pastures, manure‐applied fields, and directly from feedlots.

#### Approach to exposure assessment: Plant‐protection products

The agency responsible for pesticide risk assessment in the United States is the US Environmental Protection Agency (USEPA). They use a tiered risk‐assessment system for environmental risk assessments in which conservative assumptions are used as inputs for simplistic models in a screening‐level risk assessment at tier I. In a tier II assessment, there are several environmental scenarios encompassing a multitude of crops and their growing regions. These scenarios define the soil characteristics and daily weather inputs for the exposure models, which are used along with the product label information and the environmental fate properties of the active substances for the crop‐ and chemical‐specific inputs. Case study 2 used a standard tier II scenario for modeling exposure. Environmental exposure estimates were modeled using the Surface Water Concentration Calculator (Fry et al. 2014). Although the USEPA exposure models give concentrations for water column, porewater and sediment, as with case study 1, we are focusing on the water column.

Over 38 million ha of land was put in corn production in the United States in 2012, accounting for 30% of the harvested cropland area (US Department of Agriculture 2014). For case study 2, the USEPA standard tier II Iowa corn scenario (US Environmental Protection Agency 2017a) was selected as representative of intense US corn production.

The standard USEPA ecological exposure assessment is based on a single 10‐ha field in which all runoff and erosion drains to a single 1‐ha, 2‐m‐deep pond. However, for our exposure scenario in which multiple fields within a catchment drain to a common water body, the USEPA Index Reservoir (US Environmental Protection Agency 2010) was implemented because this allows for a mixed‐use watershed. The index reservoir is based on an actual watershed, the Shipman City Lake located in Illinois, which is a 172‐ha catchment that drains to a surface water body of 5.26 ha surface area and a depth of 2.74 m. The exposure modeling uses the conservative assumption that chemicals from all areas in the catchment reach the water body at the same time.

A typical crop‐protection treatment regime was defined using most common practices in that area. The program consists of 12 active ingredient applications, including the most widely (by area treated) applied seed treatment, corn root worm treatment, herbicide program, and fungicide. All applications were made at the standard application rate, implementing the label buffer specified on the most conservative label (200 ft [61 m] around natural or impounded lakes and reservoirs as specified for atrazine [Syngenta 2015]). Substances were applied to the corn fields as pre‐ and postemergence herbicides, fungicidal and insecticidal seed treatments, a soil insecticide, and foliar fungicides (Supplemental Data, Table S4). Critical crop dates include emergence (25 May), maturation (24 July), and harvest (19 October) as specified in the standard Iowa corn scenario.

#### Approach to exposure assessment:Veterinary pharmaceuticals

Veterinary pharmaceuticals were considered in addition to crop‐protection products, using beef cattle as the animal receiving treatment. Analysis of US Department of Agriculture Census of Agriculture National Agricultural Statistics Service data in Zoetis (2014) indicated that western Iowa contains a high density of beef feedlot cattle as well as cropland receiving manure applications. An analysis was conducted to identify highly vulnerable watersheds based on beef cattle feedlot density, manured cropland, and climate (Zoetis 2014). This analysis identified 2 counties in western Iowa (Lyon and Sioux) that are representative of highly vulnerable landscapes, within which a single watershed was selected based on high exposure potential, characterized by land use. The total watershed was 9016 ha, consisting of 56.6% corn, 2.3% pasture, and 0.94% feedlot, with the remainder composed primarily of other agriculture and developed land. More details are in the Supplemental Data, with full details in Zoetis (2014).

Land‐use area percentages for this watershed were used within the USEPA index reservoir scenario to calculate PECs. These percentages for manured land, pasture, and feedlot were used to scale the daily PRZM runoff and erosion chemical mass loadings (which assumes cropland, pasture, and feedlot are each 100% of the watershed) simulated by an individual PRZM model run before the mass enters the water body.

To model potential transport of veterinary medicines to surface water for case study 2, it was assumed that beef cattle were treated annually with an injection of tilmicosin, a macrolide antibiotic. Subsequent excretion of the active ingredient was modeled for 14 d after treatment, assuming a 50% metabolism rate, with no degradation in the manure. Cattle were also treated annually with moxidectin as a “pour‐on” application, used for parasite control. Subsequent excretion of the active ingredient was modeled for 20 d (feedlot) or 26 d (pasture) after treatment, assuming a 61% metabolism rate, with no degradation in the manure. Runoff from manure containing moxidectin and tilmicosin was modeled from pasture, as manure applied to corn fields (Supplemental Data, Table S5), and from feedlots using the inputs listed in Supplemental Data. Collection water from feedlot lagoons was assumed to have 10% of the chemical mass and was applied to the corn fields as irrigation 4 times annually.

#### Risk characterization

An RAC was determined for each of the 12 pesticide active substances in a manner comparable to the UK wheat scenario in case study 1. Because this was a US scenario, the pesticide RAC values were typically the USEPA aquatic life benchmarks (US Environmental Protection Agency 2016), except where stated otherwise in Table 4. For the veterinary pharmaceuticals, the tilmicosin RAC was based on the assessment factors in the relevant guidance (European Medicines Agency 2005), and for moxidectin the RAC value was taken from an environmental risk‐assessment report (Fort Dodge Animal Health 1997) submitted for regulatory decision‐making. One aspect highlighted was the difference in the amount of available effects data between plant‐protection products and veterinary medicines, where the former have more comprehensive data requirements and typically smaller assessment factors. This is likely a reflection of the relative route of exposure and ecological concern where veterinary products are often fed, poured on the hide, or administered by injection to animals and residues enter the environment through excreta after metabolism in vivo versus being sprayed or directly applied to the field or crop as for pesticides.

**Table 4:** Effects data and regulatory acceptable concentrations (micrograms per liter) for US corn case study

		Primary producers					Invertebrates						Fish					
Active ingredient	Group	EC50	AF	Higher tier	AF	RAC	Acute	AF	RAC	Chronic	AF	RAC	Acute	AF	RAC	Chronic	AF	RAC
Acetochlor	H	1.34	1			1.34	8200	2	4100	22.1	1	22.1	380	2	190	130	1	130
Atrazine	H	<1	1	10	1	10^a^	720	2	360	60	1	60	5300	2	2650	5	5	5
Clopyralid	H	6900	1			6900	113 000	2	56 500	17 000	1	17 000	1 968 000	2	984 000	10 800^b^	1	10 800
Clothianidin	I	64 000	1			64 000	22	2	11	1.1	1	1.1	>101 500	2	>50 750	9700	1	9700
Flumetsulam	H	3.1	1			3.1	254 000	2	127 000	111 000	1	111 000	293 000	2	146 500	197 000	1	197 000
Glyphosate	H	11 900	1			11 900	53 200	2	26 600	49 900	1	49 900	43 000	2	21 500	25 700	1	1800
Ipconazole	F	2200^c^	1			2200	1700	2	850	10.9^c^	1	10.9	1530	2	765	0.18	1	0.18
Metalaxyl	F	6250	1			6250	28 000	2	14 000	100	1	100	130 000	2	65 000	9100	1	9100
Metconazole^d^	F	1700	1			1700	4200	2	2100	78	1	78	2100	2	1050	2.91	1	2.91
Moxidectin	VM	87	100			0.87	0.03	100	0.0003			0.0003	160	100	1.6			1.6
Pyraclostrobin	F	1.5	1			1.5	15.70	2	7.85	4.00	1	4	6.20	2	3.1	2.35	1	2.35
Tefluthrin^e^	I	>1050	1			1050	0.070	2	0.035	0.008	1	0.008	0.06	2	0.03	0.004	1	0.004
Trifloxystrobin	F	37.1	1			37.1	25.30	2	12.65	2.76	1	2.76		2.76	14.3	2	7.15	4.30
Tilmicosin	VM	84	100	41	10	4.1	57300	1000	57			57	716 000	1000	716			716

^a^ Current regulatory concentration equivalent level of concern for aquatic plants as a 60‐d average (US Environmental Protection Agency 2017c).

^b^ European Food Safety Authority 2005.

^c^ No value in US Environmental Protection Agency aquatic benchmark or associated document, taken from ipconazole European Food Safety Authority conclusion 2013a.

^d^ Data from US Environmental Protection Agency 2005.

^e^ No reference given for benchmark values, taken from tefluthrin European Food Safety Authority conclusion 2010.

AF = assessment factor; F = fish; H = human; I = invertebrate; M = macrophyte; RAC = regulatory acceptable concentration; V = vertebrate.

It was assumed that the same set of substances were applied in each year over a 30‐yr period. For calculation of chronic risk, TWAs of 21 and 60 d were used for aquatic invertebrates and fish, respectively (US Environmental Protection Agency 2017b). The methodology for summing daily RQs to indicate risk were the same as for case study 1, as was the use of the MCR and grouping into categories I, II, IIIA, and IIB to facilitate communication of the risk.

#### Results case study 2

Table 5 gives the number of days when the RQ exceeded 1 for individual chemicals for primary producers and acute and chronic risk to aquatic invertebrates and fish, together with the number of days where the ∑RQ across all of the chemicals exceeded 1 for each group. Table 3 translates these results into MCR categories. Table 5 also includes information on the duration of ∑RQ exceedances expressed as the number of times the ∑RQs exceeded 1 for a consecutive sequence of days (e.g., for 4, 21, or 60 d consecutively), as well as the longest duration of ∑RQ exceedance.

**Table 5:** . Number and percentage of total days when individual chemical risk quotient (RQ) and ∑RQ were >1 in the US corn catchment, together with the maximum RQ and consecutive days exceeding 1

	Primary producers			Invertebrate acute			Invertebrate chronic			Fish acute			Fish chronic		
	Days RQ >1			Days RQ >1			Days RQ >1			Days RQ >1			Days RQ >1		
	No.	% Total	Max. RQ	No.	% Total	Max. RQ	No.	% Total	Max. RQ	No.	% Total	Max. RQ	No.	% Total	Max. RQ
Acetochlor	575	5.25	18.19	0	0.00	0.01	0	0.00	0.81	0	0.00	0.13	0	0.00	0.08
Atrazine	361	3.29	2.21	0	0.00	0.08	0	0.00	0.42	0	0.00	0.01	1188	10.84	4.42
Clopyralid	0	0.00	0.00	0	0.00	0.00	0	0.00	0.00	0	0.00	0.00	0	0.00	0.00
Clothianidin	0	0.00	0.00	0	0.00	0.05	0	0.00	0.41	0	0.00	0.00	0	0.00	0.00
Flumetsulam	0	0.00	0.26	0	0.00	0.00	0	0.00	0.72	0	0.00	0.00	0	0.00	0.00
Glyphosate	0	0.00	0.00	0	0.00	0.00	0	0.00	0.00	0	0.00	0.00	0	0.00	0.00
Ipconazole	0	0.00	0.00	0	0.00	0.00	0	0.00	0.00	0	0.00	0.00	0	0.00	0.01
Metalaxyl	0	0.00	0.00	0	0.00	0.00	0	0.00	0.00	0	0.00	0.00	0	0.00	0.00
Metconazole	0	0.00	0.00	0	0.00	0.00	0	0.00	0.01	0	0.00	0.00	0	0.00	0.14
Moxidectin	0	0.00	0.00	48	0.44	3.18	0	0.00	0.84	0	0.00	0.00	0	0.00	0.00
Pyraclostrobin	0	0.00	0.38	0	0.00	0.07	0	0.00	0.06	0	0.00	0.18	0	0.00	0.06
Tefluthrin	0	0.00	0.00	41	0.37	9.89	49	0.45	1.25	47	0.43	11.54	599	5.47	2.49
Tilmicosin	0	0.00	0.13	0	0.00	0.01	0	0.00	0.00	0	0.00	0.00	0	0.00	0.00
Trifloxystrobin	0	0.00	0.00	0	0.00	0.00	0	0.00	0.00	0	0.00	0.00	0	0.00	0.00
∑RQ	1100	10.04	18.57	113	1.03	11.44	824	7,52	3.47	49	0.45	11.63	1980	18.07	5.92
Max. duration ∑RQ >1 (days)	177			5			115			3			279		
Days ∑RQ >1 for >1 d	1080			53			806			15			1962		
Days ∑RQ >1 for >4 d	1023			2			752			1			1908		
Days ∑RQ >1 for >21 d	754			0			510			0			1602		
Days ∑RQ >1 for >60 d	387			0			142			0			937		

The exposure profiles for the individual chemicals which drove the risk assessments were very different in this case study compared with the UK case study. The UK water body is flowing, and convective transport out of the considered portion of the water body is important when characterizing exposure. In contrast, turnover of water (i.e., water entering and leaving) is much slower in the reservoir used in the US case study, so there is limited loss of chemicals under conditions where degradation is slow. As a consequence, chemicals showed much slower dissipation after an initial pulse, and compared with the UK study there was generally a higher proportion of the total days which showed RQs exceeding 1 both for single substances and for a mixture. This is also illustrated by the larger number of times the ∑RQs exceeded 1 for a consecutive set of days (e.g., for 4, 21, or 60 d consecutively), as well as the increase in the longest duration of ∑RQ exceedances for the US case study.

For primary producers, the ∑RQs exceeded 1 on 1100 (10.04%) of the 10957 d modeled (1 January 1961 to 31 December 1990), indicating that potential further refinement, mitigation, or risk management is required. The herbicides acetochlor and atrazine were the main drivers; their individual RQs reached 18.19 and 2.21, respectively, and exceeded 1 on 575 and 361 days, respectively. All other chemicals made minor contributions to the overall RQ, with only 285 d in MCR group III (no single chemical exceeding a RQ of 1) and only 17 d in group IIIB (Table 3).

For acute risk to aquatic invertebrates, the ∑RQ exceeded 1 on only 113 d (1.03% of the total), dominated by tefluthrin and moxidectin with individual maximum RQs of 9.89 and 3.18, respectively, and exceeding 1 on 41 and 48 d, respectively. There were only 44 d in MCR group III, and of these only 3 d in IIIB, indicating the dominance of the 2 chemicals driving the risk. For chronic risk to invertebrates (using a 21‐d TWA) the RQ of 1 was exceeded on 824 d, yet the only chemical which exceeded an RQ of 1 was tefluthrin, with a maximum RQ of just 1.45 and for only 49 d. Groups IIIA and IIIB contained 307 and 468 d, respectively, indicating less dominance of 1 or 2 chemicals. Acetochlor, flumetsulam, atrazine, and clothianidin all contributed to the ∑RQ, resulting in an exceedance of 1.

The ∑RQ value for acute risk to fish was exceeded on 49 d, driven largely by a single chemical, tefluthrin, with a maximum RQ of 11.54 and exceedance of 1 on 47 d. There were only 2 d when there was a mixture risk, and again it was largely driven by tefluthrin, with minor contributions from acetochlor and pyraclostrobin being sufficient to take the ∑RQ above 1. For chronic risk to fish (using a 60‐d TWA) ∑RQ exceeded 1 on 1980 d, 18.07% of the total, with a maximum ∑RQ of 5.92. Only 2 chemicals were driving this, tefluthrin and atrazine, resulting in 416 d in group IIIA, with only 8 d in group IIIB.

For acute exposures, the longest duration of exceedances (∑RQ > 1) was 3 and 5 d for invertebrates and fish, respectively, and 177 d for primary producers (driven by the 60‐d TWA for atrazine, see footnote in Table 4). The longest duration of exceedances for chronic exposures was higher because of the use of a TWA, with 115 d for invertebrates (21‐d TWA) and 279 d for fish (60‐d TWA). The number of days where ∑RQ > 1 consecutively for more than 21 d was 0 for acute exposures to invertebrates and fish and ranged from 510 for chronic invertebrates to 1602 d for chronic fish exposures. Full results are presented in Table 5.

**Figure 2:**
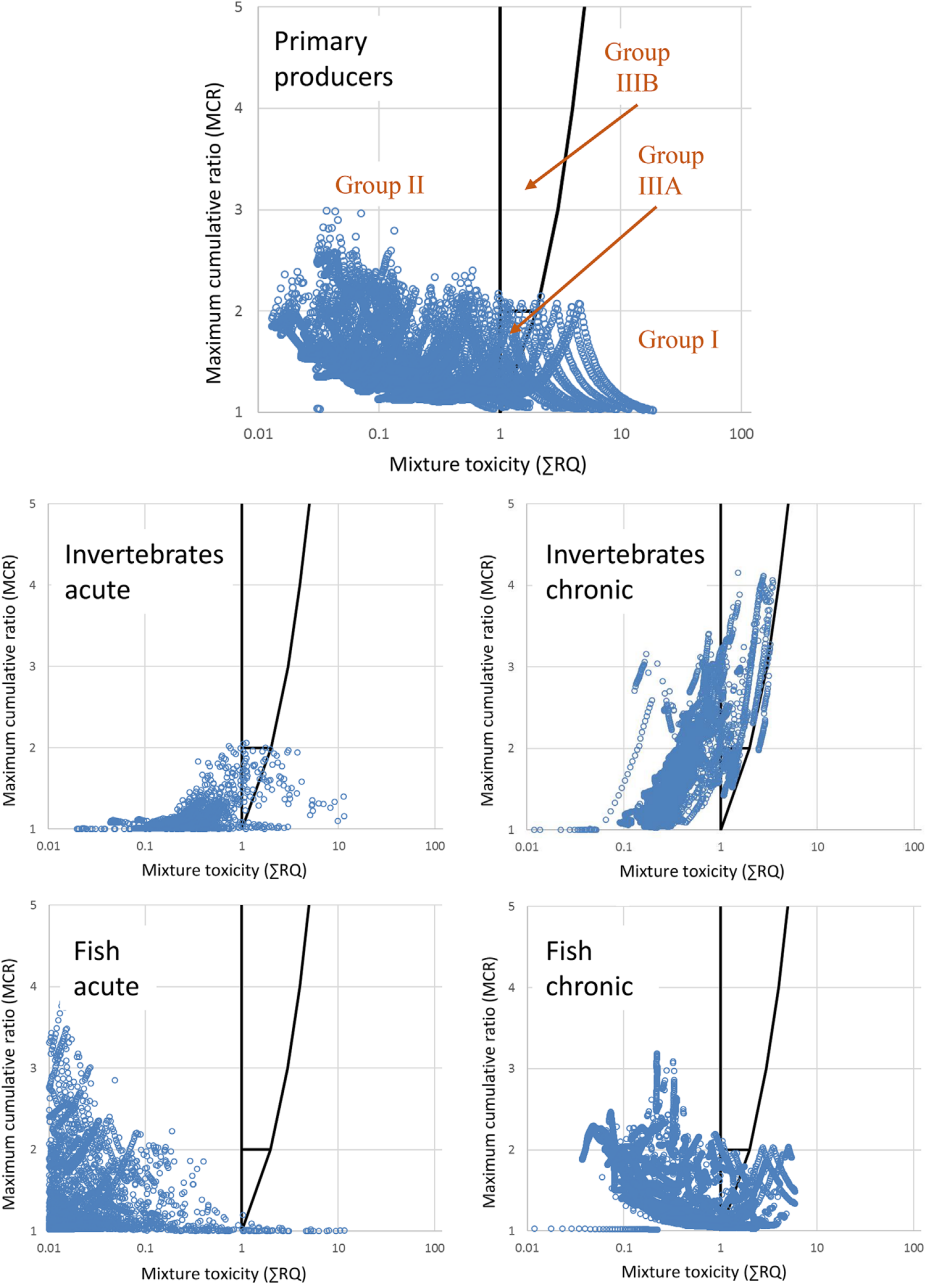
Plots of daily mixture toxicity (sum risk quotient [∑RQ], x axis) and maximum cumulative ratio (y axis) for the simulated exposure scenario of 12 plant protection products and 2 veterinary medicines used in a US catchment over 30 yr. Group I is comprised of mixtures where individual chemicals present a risk. Group II is comprised of mixtures with no risk identified. Groups IIIA (majority of risk is driven by a single substance) and IIIB (potential risk is driven by multiple components) are comprised of mixtures where only the combined effect indicates a risk. Plots are shown for primary producers (algae and aquatic plants), aquatic invertebrates (acute and 21‐d time‐weighted average [TWA] chronic), and fish (acute and 60‐d TWA chronic).

Figure 2 graphically presents the daily predicted mixture toxicity values over 30 yr for each of the taxonomic groups assessed. The topmost chart for primary producers contains the labeled MCR groups using the categories of Price and Han (2011).

## DISCUSSION

We have demonstrated the value in applying simplified, scenario‐based approaches to assessing the risks from chemical mixtures. The present case studies address agriculture in 2 continents and at the scale of a single unit and a multiunit system, and the approach allowed the consistent analysis of chemicals used for different purposes and currently assessed under different regulatory schemes (i.e., plant‐protection products and veterinary medicines). Apart from the mixture assessment step, the models we applied are those used for single‐chemical registration. Regulatory scenarios are developed to provide a prespecified vulnerability for exposure attributable to single chemicals (e.g., FOCUS 2001; Fry et al. 2014) that is associated with the stated protection goal (e.g., European Food Safety Authority 2013b). Applying these scenarios in the context of chemical mixtures reframes the problem formulation and will require reappraisal of the environmental context to deliver an appropriate level of vulnerability/protectiveness. There were some constraints in our direct application of modeling approaches aimed at single chemicals. For example, the European Union's surface water assessment is a short‐term (100‐d) calculation (FOCUS 2001) where the time window of assessment is selected according to timing of use from a total range of possibilities spanning 20 yr. It was necessary to develop a custom approach with a full 20 yr of assessment to put the analysis onto a consistent time basis for all mixture components and to investigate the range of mixtures possible as a function of variation in weather. It is notable that work is currently planned to move single‐chemical exposure assessment onto this longer‐term basis (European Food Safety Authority 2017). Current guidance on exposure modeling of veterinary medicines does not provide specific time series exposure scenarios, so the models and scenarios used for pesticides were adapted following Zoetis (2014). The Surface Water Concentration Calculator (Fry et al. 2014) model used for USEPA tier II exposure modeling in the United States directly links the model for off‐site transport of chemical to the receiving water body model. Because multiple routes of runoff entry were modeled for veterinary medicines (pasture, manured fields, feedlot), a custom step was needed to aggregate the daily mass entering the reservoir from all 3 sources before receiving water modeling was performed.

We applied a default approach of concentration addition to the effects assessment, investigating whether exposure to multiple chemicals would significantly alter the risk compared with separate assessments for each individual component of the mixture. Both case studies (edge of field and catchment scale) delivered some evidence to support considering mixtures in addition to single compounds because there were instances triggering concern for the predicted mixtures when the individual compounds would not have raised concerns in the current assessment approach. This occurred for primary producers, aquatic invertebrates, and fish in both the UK and US case studies. However, in common with other mixture toxicity studies (Belden et al. 2007), we found that a small number of chemicals were the primary drivers of instances where ∑RQ > 1 and that generally these key components of mixture toxicity were chemicals where individual risk was indicated on occasions. However, we also identified chemicals where individual RQ did not approach 1 but that made a significant contribution to mixture toxicity through frequent presence at concentrations with RQs <1 but >0.1. The signature of an individual chemical in terms of whether and how it contributes to mixture toxicity will be a function of extent of use, persistence, pathway(s) into the environment, and toxicity profile; the implication of our results is that future work could combine these factors to categorize chemicals into different characteristic contributions to mixture toxicity.

Characteristics of the receiving water body had a significant influence on assessment results in terms of both level of risk and type of risk identified. In the UK case study fish were the taxa identified most often at potential risk, driven by RQs derived from chronic RACs compared with 7‐d TWA exposures. This case study used an European Union scenario with a flowing water body where advective loss of pesticide from the system was a dominant route of dissipation. The use of a TWA reduces the RQs and may often be sufficient to demonstrate acceptable risk; failing this, a long‐term toxicity test in which the predicted, modeled exposure profile is mimicked could be conducted to link the exposure to effects. Further effects refinement could examine whether application of the concentration addition assumption is appropriate, particularly for the chronic effect endpoints (i.e., do the chemicals studied have the same mode of action or have common adverse outcomes).

The water body considered within the US exposure scenario was a reservoir with long hydraulic residence times; modeled chronic exposures were thus much more common, as were the resulting risks from single chemicals and mixtures. A generalized finding from this research is that the risk consequences of the combination of chemical use profiles and scenario characteristics can be studied in relevant detail by considering the inherent vulnerability of different taxa and the nature of potential impacts on those taxa of specific chemicals (e.g., insecticides affecting arthropods), thus helping to prioritize management decisions.

The scenario‐based approach made it possible to place the exposure assessment for 2 chemical groups with different regulatory paradigms onto a consistent basis, as illustrated for plant‐protection products and veterinary medicines in the US case study. Consistency in effects assessment was more difficult to achieve because of the different demands on data generation for different chemical types. Plant‐protection products are data‐rich with respect to ecotoxicology when compared with most animal health products. Consequently, to derive an RAC for this exercise, the assessment factors applied to the animal health products (100–1000) were large in comparison with the plant‐protection products (1–5), which could have led to the animal health products being given undue weight in the mixture risk assessment. There were instances of mixture toxicity across plant‐protection products and veterinary medicines, implying the need for better sharing of risk methodologies and risk outcomes across types of chemical. This theme is explored further in Posthuma et al. (2018) in consideration of more complex mixtures in larger catchment systems.

Our compilation of effects data highlighted a number of issues pertinent to risk assessment of chemicals and in particular mixtures. The effects data can be limiting, with the most obvious example being the disparity between the data‐rich plant‐protection products and the more data‐sparse veterinary medicines in the US scenario. This resulted in different assessment factors being applied and potentially more precaution for the veterinary medicines. However, among the pesticides there are differences in the availability of data for refinement. For example, the UK scenario indicated fluoxastrobin as the major contributor to ∑RQ for chronic risk to aquatic invertebrates; unlike some of the other chemicals, this was not based on a higher‐tier effects evaluation and so again was likely to be more precautionary. For chronic risk to fish in the United States, atrazine was a major contributor to the ∑RQ; however, the current USEPA benchmark of 5 μg/L is based on a study classified as supplemental and where the lowest‐observed–adverse effect concentration is 50 μg/L. This is a much larger range between no‐observed–adverse effect concentration and lowest‐observed–adverse effect concentration than is typical, indicating that the benchmark of 5 μg/L may be conservative and that further refinement of the effects value is a possibility.

Ecological risk assessment is geared toward protecting populations, communities, and ecosystems, rather than the individual, although an exception to this is vertebrates where no visible mortality of individuals is often the protection goal (European Food Safety Authority 2013b). At lower tiers, an assessment factor is added to single‐species laboratory acute (median lethal and effective concentrations) and, if available for the European Union, chronic (no‐observed‐effective concentration, effective concentration) values, to extrapolate to a concentration at which no effects on the community are expected. Higher tiers can involve extrapolation from laboratory toxicity data for additional species (e.g., SSDs) or community‐level studies (microcosms/mesocosms) to give concentrations at which no effects or no adverse/unacceptable effects on exposed communities would be expected. The concentration addition concept, which is widely accepted as being a conservative, default assumption for assessing the impact of chemical mixtures (European Food Safety Authority 2017), is based on single‐species approaches. Community‐level effects may depend not only on direct toxicological based effects but also on indirect ecological effects and ecological interactions (Scientific Committee on Health and Environmental Risks et al. 2012), and it is uncertain as to how, or indeed whether, these should be combined using concentration addition. Many plant‐protection products require higher‐tier tests, such as community‐level studies, to establish safe use. Without the use of higher‐tier data, therefore, a mixture assessment would likely indicate unacceptable risk because the risk from these single chemicals would already be considered unacceptable. To avoid this situation, a pragmatic approach has been adopted in the European Union (European Food Safety Authority 2013b) whereby data from both lower and higher tiers are combined in an additive risk assessment using the RACs. Comparison of risk‐assessment outcomes executed in this way with thresholds of effects in multispecies (field) tests or field ecosystems can elucidate the level of protection for this approach.

Retrospective assessment of chemical mixtures yields important information that can be used to validate modeling steps, calibrate the outcomes of prospective assessments, and determine whether any environmental impairment can be expected from, or attributed to, combinations of chemicals present in the environment. Use of monitoring data for retrospective analyses may be challenging because data exist only for sampling locations that are specifically located in space and time and only for chemicals that are specifically analyzed. Two approaches may be used for monitoring strategies of chemicals and mixtures. The first of these is targeted monitoring at a specific site or sites using prior knowledge of chemical use to indicate what to look for, such as monitoring for pesticide residues in watersheds draining from sugarcane‐growing areas in Australia (O'Brien et al. 2014). The second approach is to search monitoring databases retrospectively and determine whether there was likely to be any potential risk attributable to individual chemicals and/or mixtures. This can be done to analyze for any trends of increasing or decreasing risk (when data are available over time), and it may help to quantify the effectiveness of past mitigation measures, such as changes in the authorization of specific pesticides in reducing single‐chemical or mixture risks. Vallotton and Price (2016) illustrated this approach for pesticides in surface waters from across the United States, using results from the National Water‐Quality Assessment program of the US Geological Survey from 1992 to 2001. Using a total of 4380 samples across the United States, pesticide residues were found in 3099 and a total of 81 different pesticides were detected (average of 9 per sample, minimum of 5, maximum of 29). Hazard quotients, equivalent to the RQs discussed in the present study, and MCRs were calculated and refined based on different organism groups: fish, invertebrates, vascular (macrophytes), and nonvascular (i.e., algae) plants. Like the case studies in the present study, the retrospective analyses of Vallotton and Price (2016) allowed identification of the dominant contributors to mixtures, which were the insecticides diazinon and chlorpyrifos and the herbicides atrazine and acetochlor; interestingly, these are the same 2 herbicides giving the most concern in our US simulation, case study 2.

## CONCLUSIONS

Although the 2 case studies presented are illustrative and have limitations, the results encompass some clear patterns which relate to the study goals. First, both case studies (edge of field and catchment scale) generated evidence to support prospectively considering mixtures in addition to single compounds because there were instances across all taxa examined triggering concern for the predicted mixtures when the individual compounds would not have raised concerns in the current assessment approach. For the UK edge‐of‐field study, this only occurred between 0.18 and 2.67% of the days modeled for primary producers, invertebrates (acute and chronic), and fish (acute and chronic). This accounted for 20 to 100% of the total days when the ∑RQ exceeded 1. For the US catchment‐scale case study, mixture concerns in the absence of single‐chemical concerns occurred between 0.02 and 7.07% of the days modeled across the same taxonomic groups. This accounted for 4 to 94% of the total days when the ∑RQ exceeded 1. Second, the case studies provide insights into how often and by how much chemical exposures exceeded levels of concern either singly or in combination. Third, the case studies indicated that the relative importance of chemicals in mixtures differs and identified the chemicals that most often have an RQ >1 individually and those that may often contribute to the overall toxicity without ever exceeding an RQ of 1.

The characteristics of the receiving water body used in the exposure assessment play a key role in determining which types of substances contribute to ecological risk. Our case studies examined 2 different types of surface water: a flowing water body with significant dissipation (UK case study) and a predominantly static reservoir where aquatic degradation was the primary mechanism (US case study). The results showed that the physical–chemical properties of the substances modeled helped to define which chemicals contributed to the mixture risk in each case study.

The amount and types of data available for different components of a mixture can greatly affect the assessment factors used and thus the resulting RACs and RQs. This can have a major effect on the outcome of the assessment and indicates the difficulty in assessing risks for mixtures which contain chemicals where effects profiles have been categorized to different extents. This may result in mixture risk being driven by the compounds with the greatest uncertainty (least data) rather than the greatest toxicity.

The present approach, based on regulatory models currently used on individual chemicals, allows for the prioritization of mixtures for further investigation or management. Further higher tier effects refinements; refinement of many of the worst‐case assumptions used in the exposure modeling; and/or inclusion of more refined catchment‐scale processes would further support drawing meaningful conclusions on the risks identified in the case studies. Further considerations could include investigation of mode of action and/or common adverse outcome groups to evaluate whether concentration or response addition is appropriate or indeed whether synergy or antagonism is a potential outcome.

## Supplemental Data

The Supplemental Data are available on the Wiley Online Library at DOI: 10.1002/etc.4049.

## Disclaimer

The authors do not have any conflict of interest with the study or results. The opinions expressed in the present study are those of the authors and not their respective employers.

## Data availability

Data, associated metadata, and calculation tools are available from the corresponding author (holmesc@waterborne-env.com).

## Data Accessibility

## Supporting information

This article includes online‐only Supplemental Data.

Supporting Data S1.
