# Supplementary material for: Prospective aquatic risk assessment for chemical mixtures in agricultural landscapes
Source: Environ Toxicol Chem. 2018 Feb 12;37(3):674–89. doi: 10.1002/etc.4049 (PMC5873440; doi:10.1002/etc.4049)
Supplement: Supplementary file 1 — Supporting Data S1. [file ETC-37-674-s001.docx]

# Supporting Information

# Prospective aquatic Risk Assessment For chemical Mixtures in Agricultural landscapeS

Authors:

^†*^Christopher M. Holmes, ^‡^Colin D. Brown, ^§^Mick Hamer, ^||^Russell Jones, ^#^ Lorraine Maltby, ^††,≡^Leo Posthuma, ^‡‡^Eric Silberhorn, ^§§^Jerold Scott Teeter, ^||||^Michael St J Warne, ^##^Lennart Weltje

Running Head:

Aquatic mixture risk assessment for agricultural landscapes

**This article includes online-only Supplemental Data**

Addresses and affiliations

† Waterborne Environmental, Inc., 897-B Harrison St., Leesburg, VA, 20175 USA. E: [holmesc@waterborne-env.com](mailto:holmesc@waterborne-env.com), T: +1 (703) 777-0005

‡ Environment Department, University of York, Heslington, York, UK

§ Syngenta, Jealott’s Hill, Bracknell, UK

|| Bayer CropScience, Research Triangle Park, NC, USA

# Department of Animal and Plant Sciences, The University of Sheffield, Sheffield, UK

†† National Institute for Public Health and the Environment (RIVM), Centre for Sustainability, Environment and Health, Bilthoven, NL

≡ Radboud University, Department of Environmental Science, Institute for Wetland and Water Research, Faculty of Science, Radboud University, Nijmegen, NL

‡‡ US Food and Drug Administration, Center for Veterinary Medicine, Rockville, MD, USA

§§ Elanco Animal Health, Greenfield, IN, USA

|||| Centre for Agroecology, Water and Resilience, Coventry University, Coventry, West Midlands, UK

## BASF SE, Crop Protection – Ecotoxicology, Limburgerhof, Germany

* Corresponding author. To whom correspondence may be addressed

**Supporting Information**

## Generation of field-scale exposure estimates

Procedures used under different regulatory schemes have many commonalities, but differ in specifics according to local environmental conditions and management practices. For example, runoff and spray drift are considered as the primary routes for pesticides to enter local surface water bodies in the US regulatory scheme, whereas the EU scheme adds drainage as an additional route.

Table S1 summarizes some of the field-scale exposure methodologies in use in different parts of the world.

Most tools are developed for a single substance, with some also considering exposure to degradation products. Running a tool once for each chemical in the mixture would generate an estimate of the substances comprising the mixture of interest. Where more than one substance type is applied to a single field (e.g. veterinary medicine products via a manure application that is then treated with PPPs), maintenance of the link with existing risk assessment practice may require simulations with different modelling tools for the different substance types. For the multi-field assessment, the tool(s) needed to be run for each of the different land management units.

The unit for field-scale assessment has been dictated by the regulatory aquatic exposure framework and scenario used. In the EU, the standard field-scale size is 1 ha. In the US, the standard Tier II scenarios uses a 10 ha field size. These were used in the further assessment steps.

The receiving water body for agricultural contaminants will be dictated at the problem formulation stage. Generally, it is expected that lotic (i.e., flowing) waters will be the focus at problem formulation due to the greater potential to receive and mix inputs from multiple land management units. Most regulatory schemes for agricultural chemicals include lentic (i.e., static) water bodies in the risk assessment and this option should be selected when appropriate.

## Catchment-scale risk assessment

Some reasons that justify catchment scale for the assessment are outlined below.

1. The protection goal / effects endpoint changes with the scale. An example here would be where an area with ecological protection status occurs lower down the catchment than areas contributing mixtures of chemicals. Alternatively, lower-order water bodies may have different status in the context of environmental policies, as happens at the point where a river system falls within the definitions of the EU Water Framework Directive.
2. Chemical mixtures are more prominent at the catchment scale. Not all land uses that contribute chemicals will co-occur in space. In an agricultural context, different land uses may occur at different locations within the catchment and consideration of urban, industrial and domestic inputs alongside those from agriculture will almost always change the scale of assessment.
3. Potential for the composition of the mixture to change and any consequent interaction between components. This is a specific extension of the case above, whereby the nature of the effects may change in response to increasing complexity of the chemical mixture moving down the catchment.
4. The time-course of exposure changes. Generally peak concentrations are smaller at the catchment scale than at the field scale, but the duration of exposure tends to be longer at the catchment scale. Catchment scale assessment is required to determine how risk changes in response to this change in the exposure profile.
5. Indication of a potential risk from a field-scale evaluation. The catchment scale operates as a higher tier of assessment by incorporating more realistic diversity in land use and chemical inputs than the field scale assessment. The catchment scale assessment will incorporate chemical fate processes that occur within the receiving water body.

## Exposure scenario development

Examples of the single unit exposure scenarios are feedlots, fields, pasture, aquaculture production areas, and potentially other inputs from non-agricultural point discharges (including those being defined by the other two work groups of the SETAC Pellston® workshop (De Zwart et al. 2017; Diamond et al. 2017).

Discharges from large feed lots are typically funneled to a large lagoon. Manure is spread onto agricultural fields and water from the lagoon is used as irrigation water for agricultural fields. Agricultural fields can consider discharges from a number of sources. For instance, inputs to streams can come from spray drift, surface runoff and erosion, and from tile drainage systems located in some fields. Inputs from the field can consider applications of plant protection products to crops, to soil (e.g. seed treatments) and/or plants either by direct application or via irrigation water with veterinary medicines present in irrigation water or manure. The presence of other chemicals in irrigation water (e.g. animal hormones), can also be considered. Fields in pasture can also consider the same discharges as previously mentioned for agricultural fields. In addition, inputs resulting from manure from pastured grazing animals can be considered. Inputs from aquaculture areas and other sources can also be considered, although these may require non-standard estimation procedures, and these were not considered in the current study.

While it is possible to consider outputs in single unit scenarios from any source, certain types of outputs will rarely be considered in agricultural scenarios, including:

- Inorganic fertilizers (e.g., phosphorous, potassium, nitrogen-ammonia). Fertilizers are considered to rarely contribute as a mixture component with significant direct chemical toxicity, although they may act as a chemical stressor or enhance productivity through promotion of algal growth (Slavik et al. 2004).
- Soil particles in runoff water (although chemicals sorbed to particles are considered)
- Dissolved organic carbon in runoff water (although chemicals present in/on dissolved organic carbon are included)
- Animal excreta including endogenous hormones except veterinary medicinal products present in excreta
- Grain storage chemicals (for example, rodenticides) will rarely impact surface water but be discharged through a drain (at least in some parts of the world).
- Silage leachate from stored animal food will also rarely be of importance.

The substances typically considered in agricultural scenario include:

- PPPs (e.g., herbicides, insecticides, fungicides, plant growth regulators)
- Veterinary medicinal products (e.g., antimicrobials and other therapeutics)
- Feed additives (e.g., antimicrobials and growth enhancers)

**Derivation of Regulatory Acceptable Concentrations (RACs)**

To identify regulatory acceptable concentrations (RACs) for both case studies, we relied on the regulatory endpoints readily available in the EU and the US respectively. For the UK case study, as it was based on actual application data from growers, all the compounds were registered in the EU and had a current EU Review report from the European Commission (or if reviewed more recently an EFSA conclusion). These were used to provide the endpoints and the standard assessment factors currently used in EU from the EFSA Aquatic Guidance (EFSA 2013) applied to derive RACs, i.e. 100 for acute (EC/LC50s) fish and invertebrates, 10 for algal/plant growth (based on ErC50 wherever possible) and chronic toxicity (NOECs) to fish and aquatic invertebrates. Mesocosm data had been used to refine some assessments and where this was the case a factor of 3 was applied to the reported NOEL/NOEAL. Table 1 in the main manuscript presents the effects endpoints used in the UK case study. For the US case study, the US EPA Aquatic Life benchmarks were used, except where indicated in Table 2 in the main manuscript.

## Case Study 1: Assessment at the unit of a single field - Winter Wheat in the UK

The FOCUS R1 runoff scenario (used in this study) comprises a free-draining light silt soil with small organic matter content (5% sand, 82% silt, 13% clay, 1.2% organic carbon) coupled with a temperate climate with moderate precipitation (600-800 mm annual average rainfall, 100-200 mm annual rainfall, 6.6-10^o^C average temperature in autumn and spring), gently to moderately sloping land (2-4%) and a range of crop types including winter cereals that was simulated for the current purpose. Dates of application and actual rates are presented in Table S2. Environmental fate parameters for all compounds are given in Table S3. Half-lives for degradation in soil were selected from aerobic laboratory studies undertaken at 20^o^C and normalized to soil moisture content at pF2 (FOCUS 2001). The soil-water partition coefficients normalized to soil organic carbon content (Koc) and Freundlich exponent (nf) were selected from standard batch studies. Where the number of studies available exceeded four, standard regulatory practice was followed with the geometric mean of all available values as input to the model for degradation half-life and Koc and the arithmetic mean used for nf (EC 2014). Generally, there were two natural water/sediment values available and here the arithmetic mean was taken for degradation in the water and sediment phases.

**Case Study 2: Assessment at the small catchment unit scale – USA corn together with cattle grazing and feedlot operations**

Modeling of the USEPA Tier II Iowa corn scenario used daily weather data for 30 years from the Moline, Illinois station. The soil in the Iowa corn scenario is the Fayette silty clay loam (0.93% organic carbon, 6% slope) hydrologic group B soil. Soils in hydrologic group B have moderately low runoff potential when thoroughly wet. Water transmission through the soil is unimpeded. Group B soils typically have between 10 percent and 20 percent clay and 50 percent to 90 percent sand. Modelling inputs for the crop protection chemicals are given in Table S6.

The two counties in western Iowa (Lyon and Sioux) that are representative of highly vulnerable landscapes ranked above the 98^th^ percentile in feedlot density (n=1380), above the 99^th^ percentile in manured acre density (n=2,887), and above the 90^th^ percentile in pasture cattle density (n=3,019), however rainfall ranking (annually, March only, and October only) was in the low 20^th^ percentile (n=3110). However, if the geographic extent was constrained to only counties with moderate and high beef cattle production, the Lyon and Sioux counties would rank much higher).

Using sub-watersheds (USDA-NRCS 2006) an exposure index (based on potential for medicines in cattle manure to move to surface water) was calculated for the 42 watersheds in Lyon and Sioux counties, Iowa. From this distribution, the 90^th^ percentile watershed was selected. The feedlot land percentage of 0.094% in Iowa includes only <1000 head animal feeding operations (AFO), which are not required to be regulated under Clean Water Act and do not have NPDES discharge permits (<https://www.epa.gov/npdes>), and hence were considered as potential source of surface runoff. Concentrated animal feeding operations (CAFOs) containing greater than 1000 head are regulated under NPDES permits and are not allowed discharges with adverse effects to nearby surface waters, and were not included in the feedlot land percentage. The pasture and manure application to cropland were simulated with PRZM 5 model. The feedlot model was simulated with WINPRZM (the EU PRZM model version) which has the capability of simulating feedlot scraping (i.e., periodic manure removal from the feedlot surface) in the model. The inputs used in simulating pasture and feedlot PRZM scenarios are described by Zoetis (2014). Details of the modelling inputs are given in Tables S4-S6.

**References**

BVL. 2016. <http://www.bvl.bund.de/EN/04_PlantProtectionProducts/03_Applicants/04_AuthorisationProcedure/08_Environment/ppp_environment_node.html>

De Zwart D, Adams W, Galay Burgos M, Hollender J, Junghans M, Merrington G, Muir D, Parkerton T, de Schamphelaere K, Williams R, and Whale G. Submitted for the ET&C-Pellston mixture workshop series. Aquatic exposures of chemical mixtures in urban environments: approaches to impact assessment.

Diamond J, Dyer S, Tolls J, Snape J, Coors A, Altenburger R, Leung K, Zhang X, Koelmans A. 2017. Submitted for the ET&C-Pellston mixture workshop series. Use of Prospective and Retrospective Risk Assessment Methods that Simplify Chemical Mixtures Associated with Treated Domestic Wastewater Discharges.

European Food Safety Authority (EFSA). 2013. Guidance on tiered risk assessment for plant protection products for aquatic organisms in edge-of-field surface waters. EFSA Journal 2013;11(7):3290. 268

European Commission (EC). 2014. Assessing Potential for movement of active substances and their metabolites to ground water in the EU. Report of the FOCUS Ground Water Work Group, EC Document Reference Sanco/13144/2010 version 3, 613 pp.

FOCUS, 2001. FOCUS Surface Water Scenarios in the EU Evaluation Process under 91/414/EEC. Report of the FOCUS Working Group on Surface Water Scenarios, EC Document Reference SANCO/4802/2001-rev.2, 245 pp.

Jarvis, N.J. & Larsbo, M. 2012. MACRO (V5.2): Model use, calibration and validation. Transactions of the ASABE, 55, 1413-1423.

Slavik, K., Peterson, B. J., Deegan, L. A., Bowden, W. B., Hershey, A. E. and Hobbie, J. E. 2004. Long-term responses of the Kuparuk River ecosystem to phosphorus fertilization. Ecology, 85: 939–954.

Stone Environmental, 2016. REGDISP Spray Drift Model (v1.3). http://www.stone-env.com/news-and-insights/resource-library/detail/regdisp-spray-drift-model-v1-3

Suárez LA. 2005. PRZM-3, A Model for Predicting Pesticide and Nitrogen Fate in the Crop Root and Unsaturated Soil Zones: User’s Manual for Release 3.12.2. U.S. Environmental Protection Agency, Washington, DC, EPA/600/R-05/111.

United States Environmental Protection Agency (USEPA). 2016. Models for Pesticide Risk Assessment. https://www.epa.gov/pesticide-science-and-assessing-pesticide-risks/models-pesticide-risk-assessment#aquatic

US Department of Agriculture, Natural Resources Conservation Service, National Cartography and Geospatial Center (USDA-NRCS-NCGC). 2006. HUC12 -Watershed Boundary Dataset for 12-Digit Hydrologic Units. USDA, NRCS, Stillwater, OK. http://pubs.usgs.gov/ds/270/data/DVD-1/METADATA/Huc12.htm. Accessed July 2011.

Zoetis, 2014. Environmental Assessment for Synovex® ONE (Estradiol Benzoate and Trenbolone Acetate Extended Release Implant) Feedlot and Grass for Beef Steers and Heifers. <http://www.fda.gov/downloads/AnimalVeterinary/DevelopmentApprovalProcess/EnvironmentalAssessments/UCM409353.pdf>. Accessed May 2015.

Table S1. Examples of field-scale exposure tools used in risk assessments for agricultural chemicals in surface waters

| Pathway of input | Regulatory system | Exposure tool | Reference |
| --- | --- | --- | --- |
| Spray drift | USA | AgDrift, AGDISP, REGDISP | USEPA 2016; Stone 2016 |
|  | European Union | FOCUS calculator | FOCUS 2001 |
| Surface runoff | USA | PWC (PRZM5/VVWM) | USEPA 2016 |
|  | European Union | PRZM | Suárez 2005 |
|  | Germany | EXPOSIT | BVL 2016 |
| Subsurface drainage | European Union | MACRO | Jarvis and Larsbo 2012 |

**Table S2. Application details for the winter wheat spray program considered in the UK edge of field case study**

| Chemical name | Group | Application date | Application rate (g/ha) |
| --- | --- | --- | --- |
| Flufenacet | Herbicide | 14-Oct | 240 |
| Pendimethalin | Herbicide | 14-Oct | 1200 |
| Cypermethrin | Insecticide | 6-Nov | 25 |
|  |  | 9-Apr | 25 |
| Iodosulfuron-methyl | Herbicide | 9-Apr | 2.4 |
| Mesosulfuron-methyl | Herbicide | 9-Apr | 12 |
| Epoxiconazole | Fungicide | 14-Apr | 26 |
|  |  | 27-Apr | 300 |
|  |  | 19-May | 300 |
| Boscalid | Fungicide | 27-Apr | 230 |
| Chlorothalonil | Fungicide | 27-Apr | 500 |
|  |  | 19-May | 500 |
| Proquinazid | Fungicide | 27-Apr | 20 |
| Prochloraz | Fungicide | 19-May | 160 |
| Pyraclostrobin | Fungicide | 19-May | 56 |
| Fluoxastrobin | Fungicide | 18-Jun | 35 |
| Prothioconazole | Fungicide | 18-Jun | 70 |

Table S3. Complete exposure modeling inputs for UK wheat case study

| Chemical name | Group | Molecular weight | Vapour pressure (Pa) | Solubility (mg/L) | Kfoc (L/kg) | nf | Lab DT50 in soil (d) | DT50 in water (d) | DT50 in sediment (d) |
| --- | --- | --- | --- | --- | --- | --- | --- | --- | --- |
| Boscalid | Fungicide | 343.2 | 7.2x10^-7^ | 4.6 | 771 | 0.86 | 232 | 6 | 999 |
| Chlorothalonil | Fungicide | 265.9 | 7.6x10^-5^ | 0.81 | 838 | 0.90 | 15.7 | 0.1 | 0.1 |
| Cypermethrin | Insecticide | 416.3 | 2.3x10^-7^ | 0.009 | 121,786 | 1.30 | 31.1 | 0.1 | 6 |
| Epoxiconazole | Fungicide | 329.8 | 1.0x10^-5^ | 7.1 | 1,073 | 0.84 | 226 | 66 | 120 |
| Flufenacet | Herbicide | 363.3 | 9.0x10^-5^ | 56 | 266 | 0.90 | 30 | 54 | 81 |
| Fluoxastrobin | Fungicide | 458.8 | 6.0x10^-9^ | 2.43 | 848 | 0.86 | 101 | 34 | 163 |
| Iodosulfuron-methyl | Herbicide | 493.3 | 2.6x10^-9^ | 25,000 | 21 | 0.93 | 5 | 16 | 19 |
| Mesosulfuron-methyl | Herbicide | 503.6 | 1.1x10^-11^ | 483 | 92 | 0.91 | 45.4 | 44 | 49 |
| Pendimethalin | Herbicide | 281.3 | 1.94x10^-3^ | 0.33 | 15,744 | 0.97 | 123 | 4 | 16 |
| Prochloraz | Fungicide | 376.7 | 1.5x10^-4^ | 36 | 2,017 | 0.80 | 233 | 344 | 757 |
| Proquinazid | Fungicide | 372.2 | 9.0x10^-5^ | 0.93 | 12,870 | 0.94 | 60 | 300 | 114.5 |
| Prothioconazole | Fungicide | 344.3 | 4.0x10^-7^ | 300 | 1,765 | 0.90 | 0.5 | 1.0 | 2.2 |
| Pyraclostrobin | Fungicide | 387.8 | 3.0x10^-8^ | 1.9 | 9,315 | 0.83 | 62 | 4.9 | 21 |

Table S4. Application timing for the 12 crop protection active ingredients in US corn catchment case study

| Crop protection | Group | Application date |
| --- | --- | --- |
| chemical name |  |  |
| Acetochlor | Pre-herbicide | 1 wk before planting |
| Flumetsulam | Pre-herbicide | 1 wk before planting |
| Clopyralid | Pre-herbicide | 1 wk before planting |
| Atrazine | Pre-herbicide | 1 wk before planting |
| Clothianidin | Seed treatment | At planting |
| Ipconazole | Seed treatment | At planting |
| Trifloxystrobin | Seed treatment | At planting |
| Metalaxyl | Seed treatment | At planting |
| Tefluthrin | Soil insecticide | At planting |
| Glyphosate | Post-herbicide | 28 d after emerge |
| Pyraclostrobin | Fungicide | 65 d after emerge |
| Metconazole | Fungicide | 65 d after emerge |

Table S5. Application timing for veterinary medicines in US corn catchment case study

| Veterinary Medicine (Source) | Application Date |
| --- | --- |
| Tilmicosin |  |
| Manured cropland (solid manure) | May 8, Oct 26 |
| Manured cropland (liquid manure) | May 30, June 30, July 30, Aug 30 |
| Pasture | Apr 1 – 14 (14 day excretion) |
| Feedlot | Apr 15 – 29 (14 day excretion) |
| Moxidectin |  |
| Manured cropland (solid manure) | May 8, Oct 26 |
| Manured cropland (liquid manure) | May 30, June 30, July 30, Aug 30 |
| Pasture | Aug 30 |
| Feedlot | Apr 1 – 20 (20 day excretion) |

Table S6. Complete crop protection products exposure modeling inputs for US corn case study

| Product | Poncho VOTiVO | Vortex | Trilex | Allegiance | Force 3G | Surestart/Tripleflex | | | Aatrex | Roundup Powermax | Headline AMP | |
| --- | --- | --- | --- | --- | --- | --- | --- | --- | --- | --- | --- | --- |
| Compound | Clothianidin^3^ | Ipconazole^4,5^ | Trifloxystrobin^6,7^ | Metalaxyl^8^ | Tefluthrin^9^ | Acetochlor^10^ | Flumetsulam^12^ | Clopyralid^14^ | Atrazine^1,11^ | Glyphosate^11,15,16^ | Pyraclostrobin^17^ | Metconazole^18^ |
| Type | Insecticide | Fungicide | Fungicide | Fungicide | Insecticide | Herbicide | Herbicide | Herbicide | Herbicide | Herbicide | Fungicide | Fungicide |
| Koc (mL/g) | 123 | 2431 | 2709 | 20 | 105000 | 200 | 27 | 5 | 122 | 24000 | 9000 | 1116 |
| Aerobic Aquatic Half-Life (day) | 56.4 | 344 | 4.8 | 57.5 | 78 | 13.5 | 1521 | 148 | 159 | 87 | 58 | 465 |
| Benthic Metabolism Half-life (day) | 56.4 | 344 | 4.8 | 57.5 | 78 | 13.5 | 1521 | 148 | 159 | 87 | 58 | 465 |
| Water Column Metabolism Half-life (day) | 56.4 | 344 | 4.8 | 57.5 | 78 | 13.5 | 1521 | 148 | 159 | 87 | 58 | 465 |
| Water Reference Temperature (ºC) | 25 | 25 | 25 | 25 | 25 | 25 | 25 | 25 | 25 | 25 | 25 | 25 |
| Benthic Reference Temperature (ºC) | 25 | 25 | 25 | 25 | 25 | 25 | 25 | 25 | 25 | 25 | 25 | 25 |
| Aqueous Photolysis Half-life (day) | 0 | 0 | 0 | 0 | 0 | 0 | 0 | 0 | 0 | 0 | 0 | 0 |
| Photolysis Ref Latitude (º) | 40 | 40 | 40 | 40 | 40 | 40 | 40 | 40 | 40 | 40 | 40 | 40 |
| Hydrolysis Half-life (day) | 0 | 0 | 0 | 0 | 0 | 0 | 0 | 0 | 0 | 0 | 0 | 0 |
| Soil Half-life (day) | 120.1 | 240 | 2.4 | 35 | 27.1 | 13.5 | 51 | 11 | 60 | 47 | 30 | 220 |
| Soil Ref (ºC) | 25 | 25 | 25 | 25 | 25 | 25 | 25 | 25 | 25 | 25 | 25 | 25 |
| Foliar Half-life (day) | 0 | 0 | 0 | 0 | 0 | 0 | 0 | 0 | 0 | 0 | 0 | 0 |
| MWT | 249.7 | 333.86 | 408.37 | 279.33 | 418.73 | 269.767 | 325.29 | 192 | 215.69 | 169.07 | 387.82 | 319.83 |
| Vapor Pressure (torr) | 2.85E-13 | 3.75E-07 | 1.80E-08 | 2.48E-05 | 6.30E-05 | 4.50E-05 | 2.80E-15 | 1.02E-05 | 3.00E-07 | 9.83E-08 | 1.95E-10 | 1.58E-10 |
| Solubility (mg/L) | 327 | 7 | 0.61 | 26000 | 0.016 | 223 | 5650 | 7850 | 30 | 157000 | 1.9 | 30.4 |
| Application Date (Days Since Emergence) | -7 | -7 | -7 | -7 | -7 | -14 | -14 | -14 | -14 | 28 | 65 | 65 |
| Application Rate (kg/ha) | 0.042 | 0.00054 | 0.00118 | 0.0005 | 0.1344 | 1.03 | 0.03214 | 0.1056 | 1.122 | 1.543 | 0.1052 | 0.03946 |
| Label buffer (m) | - | - | - | - | 18.288 | 60.96 | 60.96 | 60.96 | 60.96 | 60.96 | 60.96 | 60.96 |
| Modeled buffer (m) | - | - | - | - | - | 60.96 | 60.96 | 60.96 | 60.96 | 60.96 | 60.96 | 60.96 |
| Application Type | Seed Treatment | Seed Treatment | Seed Treatment | Seed Treatment | In-furrow | Ground Spray | Ground Spray | Ground Spray | Ground Spray | Ground Spray | Ground Spray | Ground Spray |
| Application Method | Incorporate | Incorporate | Incorporate | Incorporate | Incorporate | Bare ground | Bare ground | Bare ground | Bare ground | Foliar | Foliar | Foliar |
| Ground spray, boom height | - | - | - | - | - | Low boom | Low boom | Low boom | Low boom | High boom | High boom | High boom |
| Droplet | - | - | - | - | - | Fine to Medium | Fine to Medium | Fine to Medium | Fine to Medium | Fine to Medium | Fine to Medium | Fine to Medium |
| CAM | 8 | 8 | 8 | 8 | 8 | 1 | 1 | 1 | 1 | 2 | 2 | 2 |
| Incorporation Depth (cm) | 5.08 | 5.08 | 5.08 | 5.08 | 5.08 | - | - | - | - | - | - | - |
| Application Efficiency | 1 | 1 | 1 | 1 | 1 | 0.99 | 0.99 | 0.99 | 0.99 | 0.99 | 0.99 | 0.99 |
| Spray Drift | 0 | 0 | 0 | 0 | 0 | 0.002 | 0.002 | 0.002 | 0.002 | 0.003 | 0.003 | 0.003 |

1. EPA’s fact sheet on atrazine http://www.epa.gov/ogwdw/pdfs/factsheets/soc/tech/altrazine.pdf

2. Evidence of Endocrine Disruption in Amphibians due to Agriculrual Chemicals…by Marisol Maria Gutierrez

<https://books.google.com/books?id=kNGe8xbs54EC&pg=PA24&dq=aerobic+aquatic+half-life+for+atrazine&hl=en&sa=X&ved=0ahUKEwjOh7a-2bfPAhXMaz4KHaO0BoAQ6AEINjAB#v=onepage&q=aerobic%20aquatic%20half-life%20for%20atrazine&f=false>

3. Clothianidin SANCO/10533/05 Final 18 January 2005

4. EPA Pesticde Fact Sheet Ipconzaole, September 2,004

5. EFSA Journal 2013; 11(4) 3181

6, New Registration Draft Enviornmental Fate Profile/Drinking Water Assessment, June 1999. US EPA

7. Trifloxystrobin SANCO/4339/2000 Final 7 April 2003.

8. Metalaxyl-M SANCO/3037/99-final 18 Septemer 2002

9. EFSA Journal 2010; 8(12):1709

10. Acetochlor Herbicide: New Chemical Science Chapter: DP Barcode D187737, D19031`9

11. Wauchope, R.D., T.M. Buttler, A.G. Hornsby, P.W. Augustijn-Beckers, and J.P. Burt. 1992. The SCS/ARS/CES pesticide properties database for environmental decision making. Rev. Environ. Contamin. Toxicol. 123:1-155.

12. Preliminary Ecological Risk Assessment for the Registration Review of Flumetsulam PC Code: 129016, DP Barcode: 411277, Sept. 19, 2013

13 J. M. Giddings, T. A. Anderson, L. Ws. Hall, Jr., A. J. Hosmer, R. J. Kendall. R. P. Richards, K. R. Solmon, W. M. Williams, Atrazine in North American Surface Waters, SETAC

14. Conclusion regarding the peer review of the pesticide risk assessment of the active substance clopyralid, 14 December 2005

15. Glyphosate Technical Fact Sheet, National Pesticide Information Center http://npic.orst.edu/factsheets/glyphotech.html#prop

16. Glyphosate 6511/VI/99, Jan 2002.

17. Pyraclostrobin Sanco/1420/2001-Final, 8 September 2004

18. EFSA Scientific Report (2006) 64, 1-17 Conclusion on the peer review of metconazole 13 January 2006.
